# N-Methyl-D-Aspartate Receptor-Negative Autoimmune Encephalitis in a Patient With an Ovarian Teratoma and an Associated Novel Cerebrospinal Fluid Autoantibody

**DOI:** 10.7759/cureus.16334

**Published:** 2021-07-12

**Authors:** Munir A Chaudhuri, Jason M Lucas, Mona Chandra, Michael Silver

**Affiliations:** 1 Department of Medicine, Emory University School of Medicine, Atlanta, USA; 2 Psychology, The Chicago School of Professional Psychology, Chicago, USA; 3 Department of Neurology, Emory University School of Medicine, Atlanta, USA

**Keywords:** anti-nmda receptor encephalitis, seizure, adult teratoma, unexpected malignancy, autoimmune encephalitis, paraneoplastic encephalitis syndromes, autoantibodies

## Abstract

Autoimmune encephalitis is most commonly caused by autoantibodies against N-methyl-D-aspartate (NMDA) receptors, and the malignancy most often associated with anti-NMDA receptor autoimmune encephalitis is an ovarian teratoma. Here, we describe a case of autoimmune encephalitis caused by a newly discovered cerebrospinal fluid autoantibody that has not been previously described and is not anti-NMDA receptor-mediated, which has been associated with an ovarian teratoma. It was successfully treated with high-dose corticosteroids and plasmapheresis followed by rituximab and chemotherapy (paclitaxel, ifosfamide, and cisplatin) for her teratoma.

## Introduction

Autoimmune encephalitis is caused by antibodies against neuronal cell surface or synaptic proteins. It usually presents with various neurologic or psychiatric symptoms and can occur with or without an underlying malignancy. The most widely studied type is anti-N-methyl-D-aspartate (NMDA) receptor autoimmune encephalitis. Approximately 80% of patients with anti-NMDA receptor autoimmune encephalitis are women with a median age of 21. The malignancy most often associated with anti-NMDA receptor autoimmune encephalitis is an ovarian teratoma. However, other less commonly associated malignancies include small-cell lung carcinoma, uterine and prostate adenocarcinoma, and pancreatic neuroendocrine tumors [1,2]. Here, we describe a case of autoimmune encephalitis caused by a newly discovered cerebrospinal fluid (CSF) autoantibody that has not been previously described and is not anti-NMDA receptor-mediated, which has been associated with an ovarian teratoma.

This article was previously presented as an abstract at the Society of Hospital Medicine 2020, Virtual Competition.

## Case presentation

**Figure 1:**
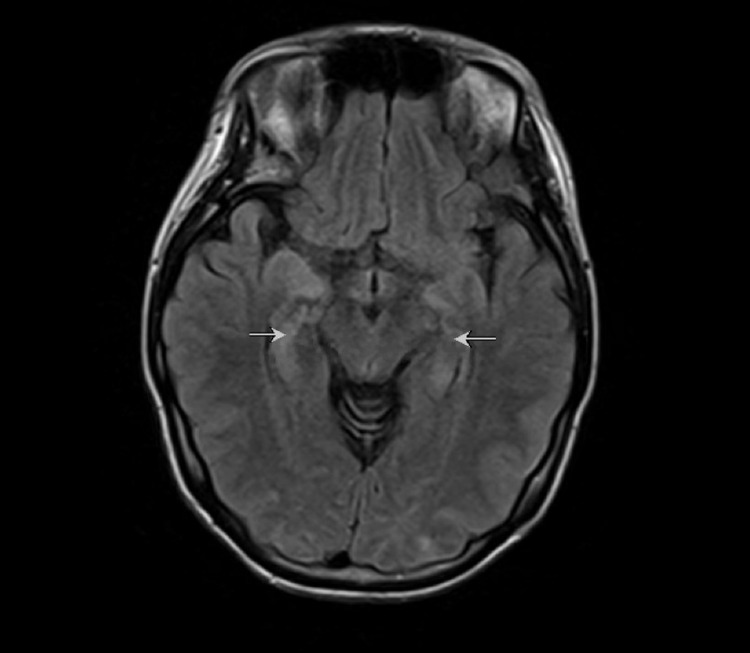
MRI brain with abnormal T2 signal in the bilateral medial temporal lobes (white arrows) concerning for autoimmune or infectious encephalitis. MRI: magnetic resonance imaging

A 29-year-old woman presented with a generalized tonic-clonic seizure and progressively worsening ataxia, memory deficits, and diplopia for months. Her past medical history included Li-Fraumeni syndrome. Her vital signs were normal except for mild tachycardia. Although she was cachectic and wheelchair-bound, there was no acute distress. She was alert and oriented and had direction-changing nystagmus, diplopia, ataxic gait, and mildly decreased strength in her lower extremities. She also had dysmetria and a bilateral postural hand tremor. The remainder of her examination was normal. CSF analysis was significant for a lymphocyte-predominant pleocytosis (CSF nucleated cell count of 7/uL [normal range: 0-5/uL] and CSF lymphocyte of 91% [normal range: 40-80%]) but was negative for a bacterial or viral central nervous system infection. Further, cytologic analysis was negative for malignant cells. The CSF NMDA receptor antibody was negative. The following autoantibodies were also tested as part of the Mayo Clinic CSF paraneoplastic autoantibody panel and were also negative: antineuronal nuclear antibody (ANNA) type 1, ANNA type 2, ANNA type 3, Purkinje cell cytoplasmic antibody (PCA) type 1, PCA type 2, PCA type Tr, amphiphysin antibody, collapsin response-mediator protein-5 neuronal, anti-glial/neuronal nuclear antibody type 1 (AGNA-1), striational (striated muscle) antibody, P/Q-type calcium channel antibody, N-type calcium channel antibody, AChR (muscle)-binding antibody, AChR ganglionic neuronal-binding antibody, and neuronal (V-G) K+ channel antibody [3]. Brain MRI revealed an abnormal T2 signal in the bilateral medial temporal lobes concerning for autoimmune or infectious encephalitis (Figure 1).

**Figure 2:**
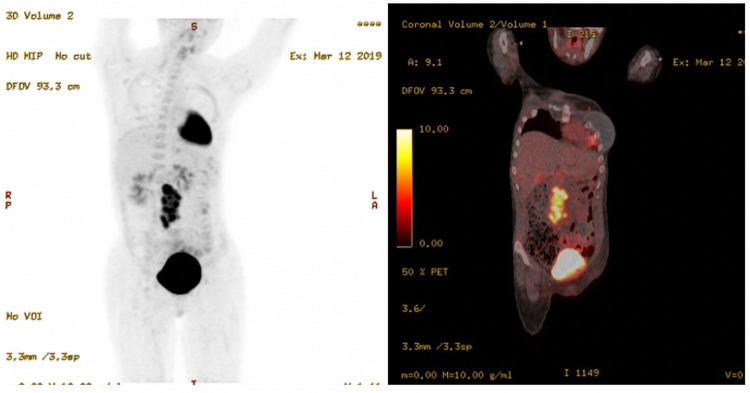
PET-CT showing interval increase in the extent and metabolic activity of the central abdominal lobulated nodal conglomerate mass, and a new hypermetabolic retroperitoneal lymph node that was confirmed by biopsy to be a metastatic immature ovarian teratoma. PET-CT: positron emission tomography-computed tomography

No spinal cord abnormalities were seen on cervical, thoracic, or lumbar MRI. A positron emission tomography scan showed hypermetabolic areas in her abdomen and a retroperitoneal lymph node concerning for malignancy (Figure 2). A biopsy was consistent with a metastatic immature teratoma. The patient was empirically treated for autoimmune encephalitis based on high clinical suspicion given her presentation, CSF profile, and the presence of the immature ovarian teratoma. Her symptoms began to improve after receiving methylprednisolone 1 g intravenously daily for five days and plasmapheresis for five sessions. However, as her symptoms did not fully resolve, she was treated with rituximab and started on chemotherapy (paclitaxel, ifosfamide, and cisplatin) for her teratoma, following which her symptoms finally resolved.

## Discussion

The first type of autoimmune encephalitis was first described in 2007 and was found to be caused by autoantibodies directed toward NMDA receptors in the CSF and was shown to be commonly associated with ovarian teratomas [1]. The most common psychiatric symptoms include visual or auditory hallucinations, psychosis, depression, and mania, while the most common neurologic symptoms include seizures, movement disorders (such as dyskinesias and rigidity), memory deficits, decreased level of consciousness, and autonomic dysfunction [4,5]. The diagnostic criteria for possible autoimmune encephalitis include subacute onset (less than three months) of neurologic or psychiatric symptoms, a new focal neurologic deficit, new-onset seizures, a CSF pleocytosis (greater than 5 cells/mm^3^), or a brain MRI showing T2 hyperintensities in the medial temporal lobes or multifocal areas involving the gray or white matter, as well as the exclusion of alternate etiologies [6].

Indirect immunofluorescence is the initial step for testing a patient’s CSF for autoantibodies. This technique uses a composite frozen section of mouse cerebellum, kidney, and gut tissue that is incubated with a patient’s sample and then washed. A fluorescein-conjugated goat antihuman immunoglobulin G is applied and neuron-specific autoantibodies are identified by their characteristic fluorescence staining patterns. Subsequently, the exact type of neuronal autoantibody is confirmed using cell-binding assay, western blot, or immunoblot. However, in rare cases, such as in our patient, when a neuron-specific autoantibody is found but further testing fails to identify it as one of the currently known autoantibodies, it is determined to be an unclassified autoantibody that has not previously been described [3,7].

The finding of a newly discovered autoantibody in this patient’s CSF that is not anti-NMDA receptor-mediated and is also associated with an ovarian teratoma is significant. This finding suggests that there may be more autoantibodies capable of causing autoimmune encephalitis that are yet to be discovered and may be associated with ovarian teratomas and possibly other malignancies that have not been commonly associated with autoimmune encephalitis. This is an important finding because even if anti-NMDA receptor autoimmune encephalitis is ruled out, it should not preclude further investigation to determine if a patient has a malignancy, particularly an ovarian teratoma. Identifying an associated malignancy is crucial because early diagnosis and treatment of an underlying malignancy in addition to immunosuppressive therapy have been shown to improve outcomes [4].

## Conclusions

Patients presenting with seizures and neurologic findings such as ataxia, memory deficits, and diplopia are commonly seen in the inpatient setting. For such patients, autoimmune encephalitis should be considered in the differential diagnosis. When clinically suspected, it is also important to investigate for the presence of an ovarian teratoma even when anti-NMDA receptor autoimmune encephalitis is ruled out. This case provides evidence that newly discovered CSF autoantibodies not directed toward NMDA receptors can also cause autoimmune encephalitis associated with an ovarian teratoma.
